# Understanding the determinants of maternal mortality: An observational study using the Indonesian Population Census

**DOI:** 10.1371/journal.pone.0217386

**Published:** 2019-06-03

**Authors:** Lisa Cameron, Diana Contreras Suarez, Katy Cornwell

**Affiliations:** 1 Melbourne Institute of Applied Economic and Social Research, University of Melbourne, Melbourne, Victoria, Australia; 2 Centre for Development Economics and Sustainability, Monash University, Clayton, Victoria, Australia; 3 World Vision Australia, Burwood East, Victoria, Australia; Tulane University, UNITED STATES

## Abstract

**Background:**

For countries to contribute to Sustainable Development Goal 3.1 of reducing the global maternal mortality ratio (MMR) to less than 70 per 100,000 live births by 2030, identifying the drivers of maternal mortality is critically important. The ability of countries to identify the key drivers is however hampered by the lack of data sources with sufficient observations of maternal death to allow a rigorous analysis of its determinants. This paper overcomes this problem by utilising census data. In the context of Indonesia, we merge individual-level data on pregnancy-related deaths and households’ socio-economic status from the 2010 Indonesian population census with detailed data on the availability and quality of local health services from the Village Census. We use these data to test the hypothesis that health service access and quality are important determinants of maternal death and explain the differences between high maternal mortality and low maternal mortality provinces.

**Methods:**

The 2010 Indonesian Population Census identifies 8075 pregnancy-related deaths and 5,866,791 live births. Multilevel logistic regression is used to analyse the impacts of demographic characteristics and the existence of, distance to and quality of health services on the likelihood of maternal death. Decomposition analysis quantifies the extent to which the difference in maternal mortality ratios between high and low performing provinces can be explained by demographic and health service characteristics.

**Findings:**

Health service access and characteristics account for 23% (CI: 17.2% to 28.5%) of the difference in maternal mortality ratios between high and low-performing provinces. The most important contributors are the number of doctors working at the community health centre (8.6%), the number of doctors in the village (6.9%) and distance to the nearest hospital (5.9%). Distance to health clinics and the number of midwives at community health centres and village health posts are not significant contributors, nor is socio-economic status. If the same level of access to doctors and hospitals in lower maternal mortality Java-Bali was provided to the higher maternal mortality Outer Islands of Indonesia, our model predicts 44 deaths would be averted per 100,000 pregnancies.

**Conclusion:**

Indonesia has employed a strategy over the past several decades of increasing the supply of midwives as a way of decreasing maternal mortality. While there is evidence of reductions in maternal mortality continuing to accrue from the provision of midwife services at village health posts, our findings suggest that further reductions in maternal mortality in Indonesia may require a change of focus to increasing the supply of doctors and access to hospitals. If data on maternal death is collected in a subsequent census, future research using two waves of census data would prove a useful validation of the results found here. Similar research using census data from other countries is also likely to be fruitful.

## Introduction

The Sustainable Development Goals aim to reduce the global maternal mortality ratio (MMR) to less than 70 per 100,000 live births by 2030 [1]. Identifying key determinants of maternal mortality and their relative importance is critical to priority setting in policy development, yet a surprisingly small number of studies have quantified the role of such determinants. That very small numbers of maternal deaths are observed in even large random samples of the population presents challenges for analyses of determinants. For example, the Indonesia Demographic and Health Survey which, owing to the absence of accurate civil registration and death reporting, is used to generate the official estimates of maternal mortality rates, surveyed 45,607 women and estimated an MMR of 359 deaths per 100,000 live births in 2012 on the basis of reports of a total of just 92 maternal deaths over the preceding five year period. It is not possible to estimate a model of the determinants of maternal mortality with such data as too few deaths are captured.

To overcome this problem, studies either: 1) examine cross-country or regional data [2–4] or combine household surveys from several countries [5], both of which can make it difficult to clearly identify determinants as there is a great deal of heterogeneity across regions/countries; 2) examine factors affecting uptake of maternal health services rather than maternal death directly [6–13]; or 3) identify cases of maternal death and then append these with a random sample of births (controls) that have not resulted in death [14–20]. Collection of the case data is costly and time-consuming and often only feasible over a limited geographic range and many of these studies are restricted to cases and controls admitted to hospitals or health centres, which are likely to be biased to particular demographics. This is where census data can prove useful. If suitable questions around the maternal status of recently deceased women are included in census questionnaires, the resultant data offer a solution to the small number of observations in other data sources and allow an examination of determinants of maternal mortality at the national and sub-national levels.

In this paper we use the 2010 Indonesian census data to identify key determinants of maternal mortality. More than 8000 maternal deaths were reported by household members in 2010. One draw-back of census data is that it often provides scant information on potential determinants of maternal mortality. The Indonesian census provides information on socio-economic status. We enrich this by merging it with village level data on the availability and characteristics of health and other community infrastructure from the 2011 Village Census (Potensi Desa, PODES). Previous studies have used the 2010 Indonesian census to calculate regional MMRs but have not used the census data to investigate the potential determinants of maternal death [21,22]. We know of only two other studies that have used population census data to study determinants of maternal mortality–in the context of Tanzania and South Africa [23–24]. Unlike this study, neither of these studies had access to detailed health service information. We estimate a model of maternal mortality and then implement a decomposition to examine to what extent differences in socio-economic status and access to health services between high MMR and low MMR provinces explain the differences in maternal mortality.

## Methods

### Data sources

The Population and Village Censuses are conducted by the Indonesian Statistical Agency (Badan Pusat Statistik, BPS). Further details on the data sources are presented in S1 Appendix. The 2010 Population Census allows identification of pregnancy-related deaths. It asks:

Has there been a death in this household since 1 January 2009? If yes, and the person who died was female and over 10 years old: Did [name] die while pregnant, during delivery or the 2 months after birth?

Restricting the sample to women aged 15–49 to match the standard definition of the MMR, the resultant group of 8075 deceased women form our cases of maternal death. To these data we appended the 5,866,791 women aged 15 to 49 years who had a live birth since 1 January 2009 and who form our sample of surviving women (controls) who we will refer to as at-risk women. While detail on the individual characteristics of the surviving women is recorded in the census, only age and information on other living household members is available for the deceased women. In the analysis below we examine the role of maternal age and household head characteristics. We also use information from the census on household housing conditions—floor type and sanitation facilities–as control variables. The 2010 census is the only Indonesian population census to date which has collected information on maternal mortality. For this reason we conduct a cross-sectional analysis.

The Village Census is a three-yearly survey of village officers in each of Indonesia’s over 60,000 villages. It collects information on village characteristics including the main source of income in the village; health services available in the village (village basic health posts; maternal and child health posts; and village birthing centres); numbers of doctors in the village; number of midwives working at the village health post; distances to the nearest hospital and health centre; and transport infrastructure. This information is collected from village staff, including health staff. In 2011 the Village Census included an additional module which collected detailed information on the characteristics of health services from interviews with health service staff. From these data we constructed variables on the number of doctors and midwives working at the health centre; indicators of whether the community health service has an inpatients service, and whether the village birthing centre (if it exists) has an inpatients service.

The Village Census aims to provide a record of infrastructure across all of Indonesia’s villages. We were able to match 95% of the villages across the Village Census and the Population Census.

### The Indonesian health sector

In Indonesia, basic primary health care, including ante-natal care, is provided at Village Health Posts (Pos Kesehatan Desa, Poskesdes). Villages also have maternal and child health posts (Pos Pelayanan Terpadu, Posyandu) which open periodically (normally once a month) and conduct basic maternal and child health checks such as monitoring child growth and providing nutritional advice. They are mainly operated by volunteers under the supervision of the sub-district Community Health Centre (Pusat Kesehatan Masyarakat, Puskesmas). Some villages have village birthing centres (Pos Bersalin Desa, Polindes). Health problems that cannot be handled at the village level are referred to the Community Health Centre which, in turn, makes referrals to hospitals.

Maternal health has been a priority area in Indonesia’s health policy agenda since the late 1980s [25]. The village midwife program (*bidan di desa*) was introduced in 1989 with the aim that a trained midwife and birth facility (*polindes*) would be placed in every village, alongside engagement of volunteers within the village (*kaders*) to promote health service utilisation [26]. The goal of a midwife in every village was largely achieved and the program has been shown to have increased access to skilled birth attendants [8]. However, concerns about quality quickly surfaced as midwife training falls short of the WHO training requirements for a skilled birth attendant and graduates have been found to score poorly in skills tests [27, 28]. Various other strategies have followed (detailed in S2 Appendix). However, despite the various iterations of national strategies, setting of targets, introduction of schemes, and large investments in expanded service provision, quality of and access to maternal health services remain at relatively low levels in Indonesia. The current reality for Indonesia is well short of the goal of universal access to health services that meet minimum standards. Reports by both the World Bank and the Indonesian National Academy of Sciences place a great deal of emphasis on the geographical inequities in access to health care services, especially higher level care, and the importance of strategies for reaching the outer islands (non-Java) [26, 29].

An analysis of six rounds of Indonesian Demographic and Health Survey (DHS) data finds that the rate of maternal health care has increased over time. In 2012 73% of women gave birth in a health facility, compared to 22% in 1986 [30]. The use of any ante-natal care increased from 81% to 95% over the same period. The study finds significant geographic variation in service utilisation with utilisation being highest in Java and Bali. Socioeconomic characteristics are a strong determinant of maternal health service utilisation, with a mother from a richest quintile household being 5.45 times more likely to give birth in a health facility than a mother from a poorest quintile household. However, inequality in access to health care was decreasing over this period with the greatest increases in utilisation being among poorer households.

### Conceptual framework

A number of authors have developed frameworks to capture the key factors contributing to maternal mortality. Most are based on a framework based on a sequence of events (pregnancy, complications thereof, and death), to which efforts to reduce maternal mortality must respond. The outcomes associated with these events are each determined by distant/contextual factors (socio-economic and cultural factors), intermediate factors (health status, reproductive status, access to health services, health care behaviour and utilisation) and unknown or unpredicted factors [31]. An extension of this framework includes health system aspects, such as referral paths for managing obstetrics complications [32] and “access” to services through all stages for a safe motherhood [33].

**Fig 1:**
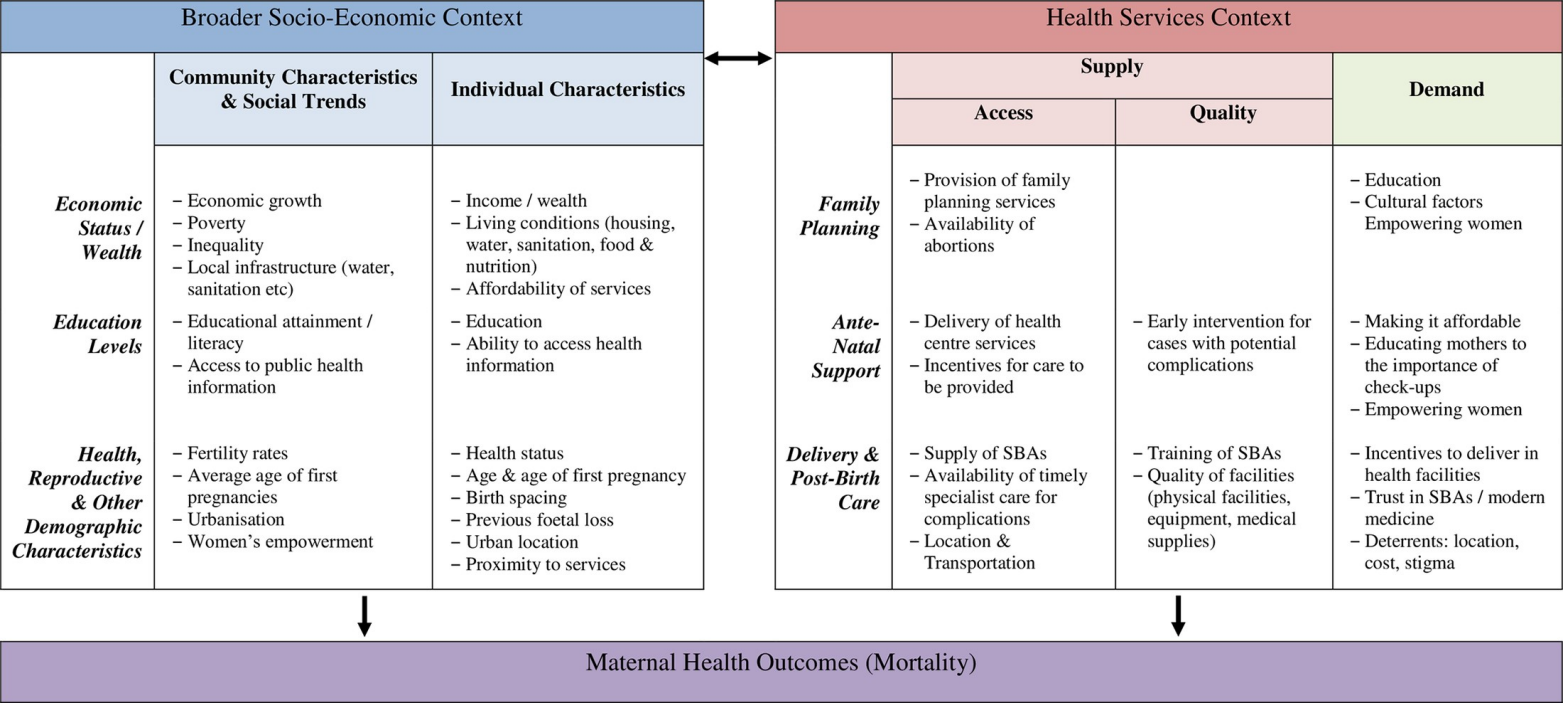
A framework for determinants of maternal mortality.

An alternative framework in which to frame maternal mortality is the “3 delays”: delay in the decision to seek care, delay in the arrival of/to care, and delay in receipt of quality care [34]. We borrow from these existing models to develop the framework shown in Fig 1 around which we structure our empirical research. We distinguish between the socio-economic and the health service factors that interact with each other and contribute to maternal mortality.

In the socio-economic context, factors such as economic status, levels of education and other health and demographic characteristics influence maternal health outcomes at both an individual and broader (national/community) level. For example, a woman’s education, income and access to household resources have effects on her health and reproductive behaviour and so will influence her age of marriage, family planning services and health care use, her ability to identify signs of risk and make use of public health information. Community economic status influences how resources are used to make good-quality care accessible to those most in need. This includes development of infrastructure (roads, transport and communications) to link women with services. While each of these factors may not directly affect the pregnancy, they set the socioeconomic context for a woman’s pregnancy and birth experience.

The health services context includes services along the pregnancy cycle–before, during and after that shape maternal health outcomes including death. The supply side is represented by the availability of services and their quality and the demand side is reflected in individual’s use of the services. For example, availability of family planning services may influence women’s decisions about the timing of their first pregnancy, spacing between pregnancies and/or access to safe abortions. Women’s empowerment, education, and cultural factors affect their ability to seek information and use it and so shapes women’s utilization for those services.

During pregnancy, the mother’s health status is determined by women’s access to antenatal care via availability of health clinics and qualified personnel that provide examinations and identify risky pregnancies. Access to specialized care and a referral system allows women to receive appropriate treatment during pregnancy and delivery, for example a planned assisted delivery at a hospital. During delivery, the key factors are who carries out the delivery, where is takes place and preparedness in case of complications. While a skilled birth attendant (SBA) should be able to deal with normal deliveries, doctors may be required in case of complications and the availability of appropriate equipment or medical supplies (e.g. blood transfusions) is key for survival in emergency situations. The use of antenatal care or use of skilled support during delivery faces several barriers on the demand side, like the affordability of the service or women’s perception of need and value.

Empirical evidence has found household income, women’s education, fertility rates, level of urbanization, household wealth and women’s health status to be strong predictors of maternal mortality [35–38]. Studies identifying determinants of maternal mortality at the individual level also find that health service access and use play a dominant role. Risk factors (e.g. mother’s age, first-births, health status) are exacerbated in areas under-served by health facilities [39] or when there are delays in seeking and reaching care [40].

While in general it has been established that access to ante-natal care improves maternal health [41], there is evidence that deliverying in a health facility is a more critical factor [42]. Birth location and available health personnel has been proven vital. In cross-countries studies, the decrease in the MMR has been associated with higher rates of SBAs [43]. There are important aspects of the provision of SBAs that impact on their effectiveness, including quality of training, proximity to clients, access to the necessary drugs and equipment, and coordination in the overall health system [44]. Research also highlights that location of birth is important to the effectiveness of SBAs. Traditional birth attendants and SBAs have comparable adverse outcomes for births that take place outside health facilities, suggesting the benefits of SBAs cannot be experienced unless the delivery takes place in a well-resourced health facility, and such care is not reached too late [45].

A systematic review of global causes of maternal deaths estimates that around 83% of maternal deaths in South-East Asia were a result of direct obstetric causes, while 17% were due to indirect causes, such as pre-existing medical conditions. Among the direct obstetric causes, haemorrhage was most common (36% of direct causes), followed by hypertensive disorders such as eclampsia (17%) [46,47]. Mortality rates are highest at the time of birth and the 24 hours postpartum [48], suggesting that interventions most effective at saving lives ought to ensure emergency care at the time of birth—the presence of skilled attendants at births and timely access to emergency obstetric care [49–52]. The barrier of distance for uptake of health services such as use of SBAs or specialized obstetric care [53] and deliveries in facilities [54,55] is well-evidenced.

### Statistical analysis

We start by estimating a multilevel multivariate logistic regression model with maternal death as the dependent variable and with a wide range of potential explanators. The full set of explanatory variables includes the age of the women (in years), urban/rural status, household head characteristics (education and employment status) which also serve as a proxy for income, housing information (sanitation access, floor type) as a proxy for wealth, village socio-economic characteristics (majority access to improved water and sanitation, main source of incomes, road type and condition) and access to and characteristics of health services (distance from village to nearest hospital and health centre (kms); whether the health centre has an inpatient facility; number of doctors and midwives working at the health centre; and within the village—number of doctors, number of midwives working at the village health post; whether the village has a birthing station; and whether the birthing station has an inpatients facility). Variables like the number of doctors or midwifes are a proxy for access to skilled birth support during the delivery. Distinguishing between the number of doctors/midwifes in the village and at health centres helps separating the effect of potentially having skilled support during delivery with or without access to a well-resourced health facility. Distance and availability of inpatient facilities are used as proxies for access to specialized care, relevant in cases of complications during delivery.

Health service utilisation data is not available in the data sources used. Although the uptake of health services is what ultimately affects maternal mortality, including health service utilisation variables as explanators is anyway problematic as the use of health services also reflects health status. Mothers in poorer health or at higher risk are more likely to utilise the services of health professionals, other things equal. Hence, the coefficient on utilisation of health services is likely to be biased downwards, and the endogeneity of these variables causes all coefficient estimates in the model to be biased and inconsistent. Methods such as instrumental variables can be used to overcome the endogeneity problem, however they rely on there being appropriate instruments that can explain utilisation but do not directly affect maternal mortality. Most variables that affect health service utilisation, for example, education levels, income etc, would be expected to directly affect maternal health. Distance to health services is often used to instrument for utilisation, which then produces results largely akin to controlling for distance to services as we do here. Sometimes studies use community averages of health utilisation to lessen the endogeneity problem but to the extent that community health service utilisation reflects village health conditions, the endogeneity problem persists.

We illustrate the predictive power of the model by calculating the predicted probability of maternal death for women with differing circumstances. We do this by substituting the characteristics of different types of women into the estimated regression equation. The role of possible confounding of impacts of observable and unobservable variables due to the cross-sectional nature of the data is explored through the inclusion of regional fixed effects. The regions are defined in line with Indonesia’s main island groupings–Java/Bali; Sumatra; Sulawesi; Kalimantan; East and West Nusa Tenggara, Maluku and Papua. The region fixed effects absorb the effect of non-time-varying unobservable regional characteristics. Any unobserved regional characteristics which are correlated with, for example, access to health care, and the probability of maternal death will bias the estimates of the effect of health care on maternal death. By absorbing the effect of unobserved non-time-varying regional characteristics, the regional fixed effects reduce the probability of the estimates being biased. Further, the sensitivity of the results to the inclusion of the fixed effects provides a sense of the extent to which the results may be driven by confounding of observable and unobservable variables.

We then conduct a Blinder-Oaxaca decomposition [56, 57] to investigate the extent to which differences in demographics and access to health services drive the differences between low maternal mortality provinces and high maternal mortality provinces. Specifically, the decomposition allows us to examine to what extent maternal mortality in the poorer performing provinces (those with a higher MMR than the national MMR- *high MMR*) would improve if health access was similar to that in the better performing provinces (MMR below the national MRR- *low MMR*). To do this we separate the sample into those with above and below average maternal mortality. We then estimate a parsimonious model which includes all statistically significant health service variables plus variables widely thought to be key in the Indonesian context, such as the number of midwives, see S2 Appendix. This parsimonious model is estimated over the sample of observations from provinces which have a MMR higher than the national MMR. The decomposition procedure then substitutes the means of the variables in the better performing regions into the estimated relationship between the variables and maternal mortality for the poorer performing regions and so predicts the MMR if these provinces had the same characteristics as the better performing provinces. The difference between the actual MMR in the higher MMR provinces and the predicted MMR for these provinces using all the characteristics of the lower MMR provinces is called the “Total Explained Component”.

All statistical analysis is conducted using Stata, version 15.0. Standard errors are clustered at the village level. The decomposition is conducted using the Oaxaca command in STATA with the logit option.

### Ethics statement

As this study uses secondary data which were fully anonymized before they were accessed, no ethics applications were submitted.

## Results

**Fig 2:**
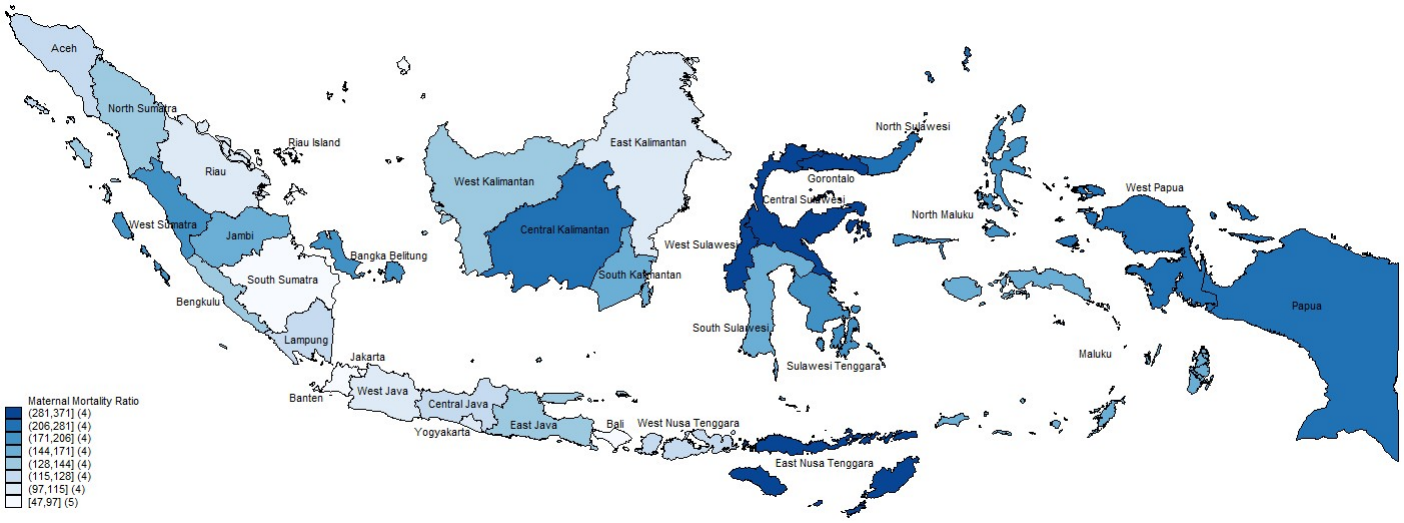
Maternal mortality ratio by province (per 100,000 live births). This map was produced by the authors using the shape file of Indonesian administrative borders available at [58].

The national MMR implied from the census data of 8075 maternal deaths and 5,866,791 live births, is 137 deaths per 100,000 live births. Fig 2 shows the variation in MMRs across the Indonesian archipelago as calculated from the census. MMRs are lower in the less-remote and more economically-developed regions of Java and Bali.

**Fig 3:**
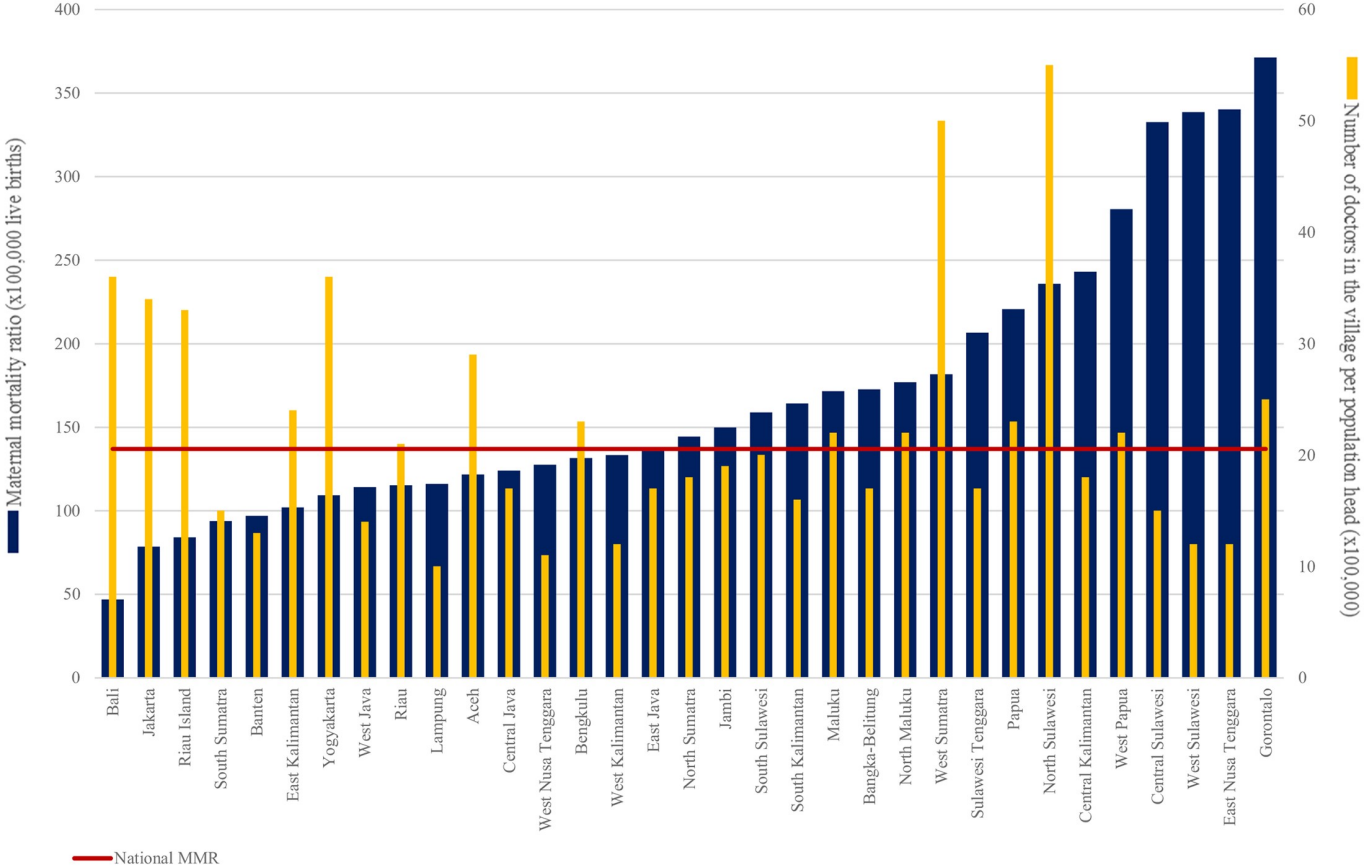
Ranking of provinces by maternal mortality ratio (x 100,000 live births).

Fig 3 ranks the provinces by their provincial MMR from the province with the lowest MMR to the highest MMR and shows the national MMR (red line) and also the average number of doctors in the village per head of village population. Bali has the lowest provincial MMR of 47. All of Java and islands to the west have rates below 200 per 100,000 live births. Gorantalo province in the north of the Outer Island of Sulawesi has the highest provincial MMR, more than seven times higher than that of Bali at 371 deaths per 100,000 live births. Very high rates are also observed in other Outer Island locations. Doctors per head of population are generally higher in the low MMR provinces and lower in the very high MMR provinces (with West Sumatra and North Sulawesi being notable exceptions). Table A in S3 Appendix presents the means of other key variables—health services, education levels and livelihoods—by province.

Table 1 provides descriptive statistics for our analysis sample. After dropping observations for which explanatory variables on health service access were missing, our final analysis sample consists of 5,567,029 women of reproductive age. Most women come from households where the household head is engaged in some form of work for income or family gain (93%). Education levels are low with 44% of household heads having attended no higher than primary education. Agriculture is the main source of income in the majority (63%) of communities. Twenty-three percent of households have rudimentary floor material (dirt, bamboo or wood) and 18% do not have a toilet. Eleven percent come from communities where open defecation is the norm. Road quality and access is limited for only a small proportion of women (4% live in villages where the widest road is unpaved, and 3% where the road to the village cannot be passed year round).

**Table 1:** Descriptive statistics for analysis sample and separately for low and high MMR provinces.

Sample:	All Provinces		Low MMR Provinces (Provincial MMR below the national MMR)		High MMR Provinces (Provincial MMR above the national MMR)		Difference between low and high MMR provinces (Low—High)	
	Mean	Std Dev	Mean	Std Dev	Mean	Std Dev	Diff	P-value
Maternal death rate	0.00133	0.03639	0.00115	0.03388	0.00192	0.04376	-0.00077	0.00
Woman’s Age (years)	28.47	6.27	28.43	6.24	28.57	6.35	-0.14	0.00
**Household Characteristics**								
Household Head’s Highest education level attained: ^a^								
Primary school (%)	34	0.47	35	0.48	29	0.46	6	0.00
Junior high school (%)	18	0.38	17	0.38	18	0.39	-1	0.00
Senior high school or above (%)	38	0.48	37	0.48	38	0.49	-1	0.00
Household head is employed (%) ^b^	93	0.25	93	0.26	94	0.24	-1	0.00
Urban (%) ^b^	52	0.5						
Poor quality floor (%) ^b^	23	0.42	55	0.5	40	0.49	15	0.00
Doesn’t have a toilet (%) ^b^	18	0.38	19	0.39	35	0.48	-16	0.00
**Village Characteristics**			16	0.37	22	0.42	-6	0.00
Most households have unimproved water source (%) ^b^	43	0.65	43	0.64	43	0.66	0	0.00
Most households do not have a toilet (%) ^b^	11	0.32	11	0.31	13	0.34	-2	0.00
Mains source of income–agriculture (%) ^b^	63	0.48	62	0.49	69	0.46	-7	0.00
Widest road surface is unpaved (%) ^b^	4	0.19	3	0.16	7	0.25	-4	0.00
Main road cannot be passed all year round (%) ^b^	3	0.17	2	0.14	7	0.25	-5	0.00
**Health services**								
Distance to the nearest hospital (10 kms)	1.23	1.75	1.07	1.57	1.77	2.18	-0.7	0.00
Distance to the nearest health centre (10 kms)	0.29	0.52	0.26	0.47	0.39	0.66	-0.13	0.00
No. doctors working at health centre (count)	2.77	2.39	2.87	2.57	2.42	1.59	0.45	0.00
No. midwives working at health centre (count)	10.59	7.67	10.96	7.59	9.32	7.81	1.64	0.00
Health centre has inpatients (%) ^b^	55	0.5	55	0.5	58	0.49	-3	0.00
No. of doctors living in the village (count)	2.71	7.13	3.06	7.84	1.55	3.65	1.51	0.00
No. of midwives working in the village health post (count)	0.43	0.7	0.42	0.66	0.44	0.8	-0.02	0.00
Village has a birthing station (%)^b^	30	0.46	29	0.45	31	0.46	-2	0.00
Birthing station has an inpatients facility (%) ^b^	6	0.24	6	0.23	8	0.27	-2	0.00
Village has a maternal and child health post (%) ^b^	100	0.06	100	0.04	99	0.12	1	0.00
Number of observations	5,567,029		4,288,855		1,278,174		5,567,029	

^a^ For household head’s education, the reference category is no education or incomplete primary school.

^b^ The reference category is the complement category, for example for urban the reference is rural and for poor quality floor the reference is a quality floor.

Access to health services and health personnel vary substantially across the archipelago. The average woman lives 12 kilometres from a hospital and 3 kilometres from a health centre. Average distances to a hospital vary widely from 0.5 kilometre in Jakarta to 29.0 kilometres in Central Sulawesi (see Table A and Figure A in S3 Appendix). Health centres on average have 10.6 midwives and 2.8 doctors. Again this is highly variable with the median number being two doctors and the highest 60. Almost all villages have a health post but many are not staffed by a midwife (average number of midwives is 0.43). Thirty percent of villages have a birthing station, but only 6% have an inpatient facility.

### Determinants of maternal mortality

Table 2 reports adjusted odds ratios and 95% CIs from the multilevel multivariate logistic regression model including the full range of control variables. Controlling for other factors, the probability of maternal death increases, at a slightly increasing rate, with the age of the mother. Likelihood of maternal death is negatively associated with the education of the household head and the head being employed. An at-risk woman from a household whose head has completed secondary education is 63% less likely to die from maternal causes than a woman from a household whose head has no education.

**Table 2:** Logistic regression results. Dependent Variable = Maternal Death (0/1).

VARIABLES	odds ratio (adjusted)	95% CI
**Woman’s characteristics**		
Age (years)	1.027	1.001–1.053
Age squared	1.000	1.000–1.001
**Household Head’s characteristics**:		
Highest level of education attained: ^a^		
Primary school	0.656	0.616–0.698
Junior high school	0.446	0.410–0.484
Senior high school or above	0.368	0.340–0.398
Employed ^b^	0.611	0.566–0.659
**Household Characteristics**		
Urban ^b^	1.013	0.943–1.088
Poor quality floor ^b^	1.024	0.966–1.086
Doesn’t have a toilet ^b^	1.064	1.000–1.132
**Village Characteristics**		
Most households have unimproved water source ^b^	1.002	0.964–1.041
Most households do not have a toilet ^b^	0.981	0.910–1.059
Main source of income—agriculture ^b^	1.011	0.934–1.095
Widest road surface is unpaved ^b^	1.017	0.895–1.157
Main road cannot be passed all year round ^b^	1.063	0.928–1.218
**Health Service Access**		
Distance to the nearest hospital (10 kms)	1.039	1.023–1.056
Distance to the nearest health centre (10 kms)	0.989	0.943–1.038
No. doctors working at health centre (count)	0.968	0.948–0.988
No. midwives working at health centre (count)	0.997	0.994–1.001
Health centre has inpatients ^b^	0.991	0.941–1.044
No. of doctors living in the village (count)	0.990	0.984–0.997
No. of midwives working in the village health post (count)	0.952	0.913–0.992
Village has a birthing station ^b^	0.976	0.918–1.038
Birthing station has an inpatients facility ^b^	0.918	0.823–1.024
Constant	0.00130	0.000865–0.00197
Number of 0bservations	5,567,029	

Notes: Standard errors are clustered at the village level.

^a^ For household head’s education, the reference category is no education or incomplete primary school

^b^ The reference category is the complement category, for example for urban the reference is rural and for poor quality floor the reference is non-poor quality floor.

Health service access strongly reduces the risk of maternal death. Every extra 10 kms a woman is from the nearest hospital is associated with a 3.9% increase in the likelihood of maternal death. Although distance to a health centre is not associated with maternal death (adj. OR 0.989, 95% CI 0.943–1.038), each additional doctor at the health centre reduces the probability of maternal death by 3.2%. Additional midwives working at health centres are not associated with a decreased risk of maternal death but the number of midwives working in the village at the village health post is protective, reducing the likelihood of maternal death by 4.8%. The number of doctors in the village is a strongly statistically significant determinant, although the magnitude of the effect is small (adj. OR 0.990 95% CI 0.984–0.997). Robustness results reported in Table C in S3 Appendix show that the results when regional fixed effects are included are very similar to the model above.

To illustrate the magnitude of these results, consider two 22 year old women whose household and village characteristics are very different. The first woman lives in a neighbourhood typical of Jakarta. She lives with her husband who has completed secondary school and is employed. Their house has a tiled floor and a toilet, with water piped to the house. Most people in their neighbourhood have similar quality housing, the roads are all paved and accessible year round. There is a hospital one kilometre away and she lives next to a health centre which has an inpatient service, and is staffed by five doctors, and eight midwives. In addition, eight doctors live in her neighbourhood. The predicted probability of maternal death for women in these circumstances is 51 deaths per 100,000 live births.

The second woman lives in a small agricultural village in Papua with unpaved roads that wash out in the wet season leaving the village inaccessible. She lives in a house with no toilet, a dirt floor and an unprotected water source like most others in her village. Her husband is not working and has only completed primary school. There are no health facilities in her village. The nearest hospital is 14 kilometres away, and it is 3 kms to the nearest health centre where there is an inpatients service with one doctor and two midwives. There is no health post, no doctors in her village and no birthing station. For the woman in these circumstances the predicted probability of maternal death is 345 per 100,000. The woman in Papua is more than six times more likely to experience a pregnancy-related death than the woman in Jakarta.

### Factors contributing to the difference between high MMR and low MMR provinces

**Fig 4:**
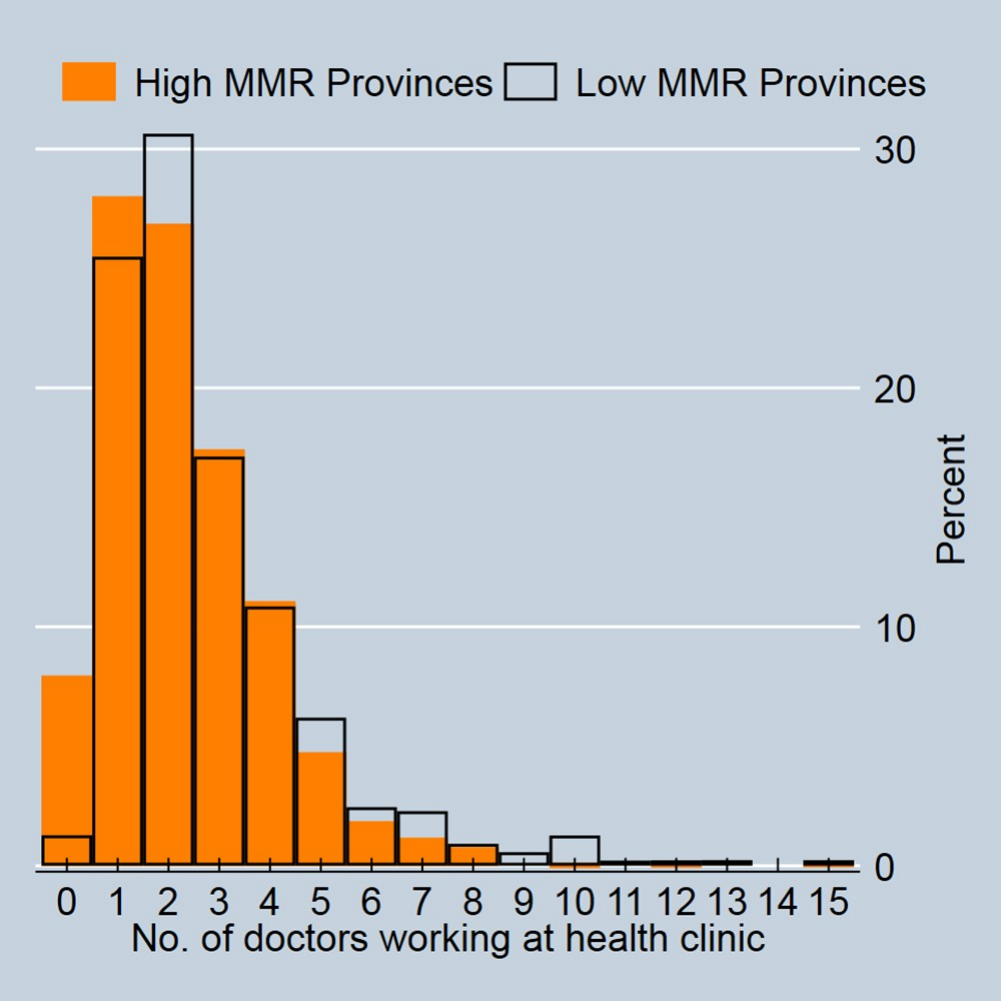
Distribution of doctors working at health clinics.

Descriptive statistics on key variables for low and high maternal mortality provinces are provided in Table 1. Health service availability is worse in higher maternal mortality provinces, particularly with respect to distance to hospitals (10.7 kms versus 17.7 kms). Although the average number of doctors differs only slightly (2.9 versus 2.4), Fig 4 shows that the distribution of doctors varies markedly with high MMR provinces having a much greater number of clinics with zero doctors. The vertical axis shows the percentage of clinics (in high MMR or low MMR provinces) which have the number of doctors shown on the horizontal axis. For example, approximately 8% of clinics in high MMR provinces have no doctors working there versus only about 1% of clinics in low MMR provinces. The modal number of doctors working at clinics is two in low MMR provinces and one in high MMR provinces.

Table 3 presents the estimation results for high MMR provinces that underlie the decomposition. This parsimonious model includes mother’s age, household head characteristics and key health service characteristics.

**Table 3:** Logistic regression. Dependent variable–Maternal death (0/1). High Maternal Mortality Rate Provinces.

VARIABLES	odds ratio (adjusted)	95% CI
**Woman’s characteristics**		
Age (years)	1.011	0.967–1.057
Age squared	1.001	1.000–1.001
**Household Head’s characteristics**:		
Highest level of education attained: ^a^		
Primary school	0.785	0.705–0.875
Junior high school	0.538	0.469–0.618
Senior high school or above	0.427	0.376–0.485
Employed ^b^	0.583	0.508–0.670
**Health Service Characteristics**		
Distance to the nearest hospital (10 kms)	1.039	1.019–1.059
No. doctors working at health centre (count)	0.918	0.887–0.951
No. midwives working at health centre (count)	0.996	0.989–1.002
No. of doctors living in the village (count)	0.980	0.962–0.998
No. of midwives working in the village health post (count)	0.927	0.857–1.003
Village has a birthing station ^b^	0.998	0.898–1.109
Constant	0.00230	0.00114–0.00463
Number of observations	1,278,174	

Notes: Standard errors are clustered at the village level.

^a^ For household head’s education, the reference category is no education or incomplete primary school.

^b^ The reference category is the complement category, for example head is not working.

Table 4 reports the decomposition results. The maternal mortality ratio in the low-performing provinces (191 per 100,000 pregnancies) is 66 percent higher than in the high-performing provinces (115 per 100,000 pregnancies). The explanatory variables explain 23.2% of this gap. The average age at which women give birth only explains a very small percentage (1.7%) of the difference in MMRs. If educational attainment and employment rates in high MMR provinces became more like low MMR provinces the analysis predicts that MMRs would *worsen* by a small amount (0.2% and 1% respectively). Almost all the explained difference is accounted for by differences in access to health services. The number of doctors working at the sub-district health centre and in the village are the most important contributors (explaining 8.6% and 6.9% of the gap respectively), followed by access to a hospital (explaining 5.9% of the gap). The number of midwives working at the health centre and at the village health post and whether the village has a birthing station are not significant contributors to the gap.

**Table 4:** How much of the difference in MMRs between higher and lower MMR provinces can be explained by their differing observed characteristics?

Maternal Mortality ratio per 1000 women	MMR	95% CI	
Provinces Below the Mean	1.149	1.115–1.183	
Provinces above the Mean	1.912	1.835–2.002	
			Percentage difference
MMR Raw Difference between high and low performing provinces	-0.769	-0.860 – -0.679	66.41%
Total explained component	-0.1786	-0.226 – -0.131	23.21%
Number of observations	5,567,029		
	Contribution	95% CI	Percentage contribution
**Contribution to the explained differences**			
Woman’s Age (years)	-0.013	-0.017 – -0.0095	1.74%
Head’s Education ^a^	0.00179	-0.00989–0.0135	-0.23%
Head Employed ^b^	0.00830	0.00533–0.0113	-1.08%
Health Services ^c^	-0.175	-0.219 – -0.132	22.78%
**Breakdown of contribution of health services**			
Distance to the nearest hospital (10 km)	-0.0457	-0.0732 – -0.0181	5.93%
No. doctors working at health centre	-0.0658	-0.0940 – -0.0377	8.56%
No. midwives working at health centre	-0.0122	-0.0319–0.0076	1.58%
No. of doctors living in the village	-0.0531	-0.0938 – -0.0124	6.90%
No. of midwives working in the village health post	0.00145	-0.00229–0.00519	-0.19%
Village has a birthing station (POLINDES)^b^	0.00004	-0.00249–0.00257	-0.005%

Notes: The percentage shown for the raw difference is the percentage difference between the mean MMR for below the mean and above the mean provinces. The other percentages shown are the contribution to the total raw difference. Age contains age and age-squared.

^a^ Head’s education contains all indicators of education level.

^b^ The reference category is the complement category, for example head is not working.

^c^ Health access contains distance to the nearest hospital, number of doctors and number of midwives working at the health centre, number of doctors in the village, number of midwives working at the village health post and whether the village has a birthing station.

## Discussion and conclusions

The national MMR implied from the census data is lower but not so different from the World Bank estimate for 2010 of 165 deaths per 100,000 live births [59] which is much lower than that calculated from the 2012 DHS of 359 per 100,000 live births [60]. Calculating an MMR for Indonesia is complicated as there is not reliable civil registration and death reporting and differing data sources and modelling assumptions produce very different results. Further, estimates using methods that allow an examination of trends over time differ wildly—declines in the MMR of 8% (using estimates from the DHS) and 52% (Maternal Mortality Estimation Inter-Agency Group modelling). The challenges of calculating an accurate MMR for Indonesia, and using census data more generally, are discussed in more detail in S4 Appendix.

There is great variation in maternal mortality ratios and access to health services across Indonesia. In densely populated Java-Bali (and a small number of other provinces) maternal mortality ratios are considerably lower than in other areas. Our results show that individual socio-economic status has a strongly protective effect, consistent with the previous literature [5, 10, 12], but explains very little of the difference in maternal mortality rates across provinces. Health service access, particularly to doctors and hospitals, explains a large part of the difference in maternal mortality, consistent with assistance by a health professional previously being found to reduce the risk of maternal death [14]. Although in Java the average density of midwives has been found to be a strong determinant of assistance by a health professional during birth [12] and distance to a health centre found to be a determinant of maternal death (for women who were assisted by a health professional) [14], we find that health services provided at the village level, including the number of midwives at village health posts, and the number of midwives at sub-district community health centres are not significant explanators of the difference between high and low MMR provinces. This is likely due to the widespread access to these services throughout Indonesia, in part due to the village midwife program.

Our results show that investment in hospitals and doctors in the Outer Islands would significantly reduce maternal mortality. Indonesia has the lowest doctor-population ratio in South-East Asia [61] and, unlike some other countries in the region such as Thailand, where the provision of skilled birth attendants was followed by increased medical facility capacity and which experienced rapid downward trends in maternal mortality, in Indonesia not all health centres can provide basic obstetric care [62]. Doctors in Indonesia are disproportionately clustered in urban centres on Java, and are often inaccessible to the poor [8, 30]. If the same level of access to these services as is available in Java-Bali was provided to the low-performing regions in the Outer Islands of Indonesia, our model predicts that the gap between maternal mortality ratios across these regions would drop by just over 20%. That is, in the provinces in which the maternal mortality ratio is currently above the mean of 191 deaths per 100,000 pregnancies, we would expect 44 deaths to be averted per 100,000 pregnancies. Policy prescriptions are however complicated by the observation that although maternal mortality ratios are high in the more far-flung provinces, due to its large population, Java/Bali accounts for 46% of all maternal deaths in Indonesia (Table A and Figure B in S3 Appendix).

The great strength of the census data is that they capture so many maternal deaths. They also have some weaknesses. First, there is likely to be some underreporting of maternal deaths by respondents in the census; it does not provide information on births for unmarried women; and it does not identify at risk women whose pregnancy did not progress to a live birth. These omissions could bias our results if the relationship between the household and village characteristics and maternal mortality differ for these cases. Hence the results presented here should be taken to apply to married mothers whose pregnancy progresses to a live birth. Second, remote communities are more likely to have missing values in the Village Census. As village level health care is likely important in remote areas as other sources of medical care are scarce, our results may understate the average role of village level health care. Finally, as information on maternal mortality is only available in the 2010 census, we are restricted to using a single cross-section of data so it is possible that unobserved household and village characteristics confound some estimates, although the stability of the estimates when region fixed effects are included suggests this is not the case. Future research will be able to further explore this issue by examining changes over time if maternal death questions are included in the 2020 census.

Notwithstanding these caveats, census data are likely to be a valuable source of information in many countries and, when merged to community level data on health and other facilities, have the potential to significantly enhance our understanding of how to reduce the occurrence of maternal death.

## Supporting information

S1 Appendix Additional information about the data. (PDF)

S2 Appendix Indonesian policy context. (PDF)

S3 Appendix Additional results. (PDF)

S4 Appendix Indonesia’s maternal mortality ratio. (PDF)

S1 Checklist STROBE statement. (PDF)
