# Supplementary material for: Understanding the determinants of maternal mortality: An observational study using the Indonesian Population Census
Source: PLoS One. 2019 Jun 3;14(6):e0217386. doi: 10.1371/journal.pone.0217386 (PMC6546237; doi:10.1371/journal.pone.0217386)
Supplement: S1 Appendix — (PDF) [file pone.0217386.s001.pdf]

S1 Appendix. Additional information about the data

The 2010 Indonesian census was conducted in May 2010. Where there had been a death of a female household member aged 10 or above since January 1st, 2009, further questions were asked about whether the death occurred while the woman was pregnant, during delivery or in the two months after birth. These questions allow deaths to be classified as pregnancy-related or not - according to the ICD-10 classification: "the death of a woman while pregnant or within 42 days of termination of pregnancy, irrespective of the cause of death (obstetric and non-obstetric)" [1]. Restricting the sample to women aged 15-49 to match the standard definition of the MMR, the resultant group of 8075 deceased women form the sample of maternal deaths.

The census also collected details on each individual household member currently living in the household. For female members aged 10 and above who had ever been married, whether they had delivered a live birth since 1 January 2009 is reported. The 5,866,791 women aged 15-49 who had a live birth since 1 January 2009 form our sample of surviving women. The census questions relating to maternal death are only asked of all women aged 10 and above, while the questions relating to births is only asked of women aged 10 and above who have ever been married.

The Village Census is a three-yearly survey of village officers in each of Indonesia's over 60,000 villages. It collects information on village characteristics including the main source of income in the village; health services available in the village (village basic health posts; maternal and child health posts; and village birthing centres); numbers of doctors in the village; number of midwives working at the village health post; distances to the nearest hospital and health centre; and transport infrastructure. This information is collected from village staff, including health staff. In 2011 the Village Census included an additional module which collected detailed information on the characteristics of health services from interviews with health service staff. From these data we constructed variables on the number of doctors and midwives working at the health centre; indicators of whether the community health service has an inpatients service, and whether the village birthing centre (if it exists) has an inpatients service.

The Village Census data set was merged onto the Population Census data using village codes. We were able to match 95% of the villages across the Village Census and the Population Census. We then dropped women outside the age range of 15-49 years and those with missing values for age (529 observations, <0.01% of the sample) and those which were outliers in terms of the number of household members (more than 29 members - 327 observations, <0.01% of the sample).

Our final analysis sample drops all observations with missing values for any of the explanatory variables in the model shown in Table 2. There were 191,360 (3.3%) observations with missing values for distance to nearest hospital; 241,272 (4.1%) for distance to nearest health centre; and 230 (less than 0.01%) missing values for number of doctors and midwives at the local health centre, whether the local health centre has an inpatient facility, whether the village has a birthing station and whether the birthing station has an inpatient facility. There were no missing values for any of the other variables, including the outcome variable, maternal death.

Table A in S1 Appendix (below) compares the characteristics of observations included and excluded from the analysis sample. It shows that observations with missing values were more likely to be in remote areas (10% urban if excluded versus 52% urban if included, 28% have a main road that cannot be passed year round versus 3% if included) and are of lower socio-economic status (22% have an education at senior high school level or higher, versus 38% if included, 58% have a poor quality floor versus 23% if included). Excluded observation also have a higher likelihood of maternal death (0.0023 versus 0.0013).

Table A. Comparison of Observations Included and Excluded from Analysis Sample

	Included		Excluded	
	N	Mean	N	Mean
Maternal Death	5,567,029	0.0013	307,837	0.0023
Age	5,567,029	28.47	307,837	27.98
**Household Head's characteristics:**				
Highest level of education attained:				
Primary school	5,567,029	0.34	307,837	0.40
Junior high school	5,567,029	0.18	307,837	0.16
Senior high school or above	5,567,029	0.38	307,837	0.22
Employed	5,567,029	0.93	307,837	0.96
**Household Characteristics**				
Urban	5,567,029	0.52	307,837	0.10
Poor quality floor	5,567,029	0.23	307,837	0.58
Doesn't have a toilet	5,567,029	0.18	307,837	0.39
**Village Characteristics**				
Most households have unimproved water source	5,567,029	0.43	307,837	0.60
Most households do not have a toilet	5,567,029	0.11	307,837	0.31
Main source of income - agriculture	5,567,029	0.63	307,837	0.93
Widest road surface is unpaved	5,567,029	0.04	307,837	0.24
Main road cannot be passed all year round	5,567,029	0.03	307,837	0.28
**Health Service Access**				
Distance to the nearest hospital (10 kms)	5,567,029	1.23	116,477	3.05
Distance to the nearest health centre (10 kms)	5,567,029	0.29	66,565	0.52
No. doctors working at health centre	5,567,029	2.77	307,607	1.56
No. midwives working at health centre	5,567,029	10.59	307,607	6.25
Health centre has inpatients	5,567,029	0.55	307,607	0.70
No. of doctors living in the village	5,567,029	2.71	307,837	0.47
No. of midwives working in the village health post	5,567,029	0.43	307,837	0.30
Village has a birthing station	5,567,029	0.30	307,607	0.29
Birthing station has an inpatients facility	5,567,029	0.06	307,607	0.06

S1 Appendix References

1 WHO. The WHO Application of ICD-10 to deaths during pregnancy, childbirth and the puerperium: ICD-MM. Geneva: World Health Organisation, 2012.
