# Supplementary material for: Understanding the determinants of maternal mortality: An observational study using the Indonesian Population Census
Source: PLoS One. 2019 Jun 3;14(6):e0217386. doi: 10.1371/journal.pone.0217386 (PMC6546237; doi:10.1371/journal.pone.0217386)
Supplement: S2 Appendix — (PDF) [file pone.0217386.s002.pdf]

S2 Appendix. Indonesian policy context

Maternal health has been a priority area in Indonesia's health policy agenda since the late 1980s [1]. The village midwife program (*bidan di desa*) was introduced in 1989 with the aim that a trained midwife and birth facility (*polindes*) would be placed in every village, alongside engagement of volunteers within the village (*kaders*) to promote health service utilisation [2]. The goal of a midwife in every village was largely achieved. However, concerns about quality quickly surfaced as midwife training falls short of the WHO training requirements for a skilled birth attendant and graduates have been found to score poorly in skills tests [3, 4].

Further initiatives were undertaken in the latter half of the 1990s with a focus on educating mothers and communities to seek antenatal care and assistance at the time of birth. This included the 1996 *gerakan sayang ibu* ("cherish mothers movement") program, which was initiated to encourage pregnant women and their families to seek antenatal care and assistance with the birth. The movement focussed on risk profiling and community efforts to reduce the risks [5]. The 1999 Maternal and Newborn Health Program and SIAGA media campaigns socialised communities about warning signs of postpartum haemorrhage, encouraged utilisation of skilled birth attendants (SBAs), delivery in a health facility and organisation of transport [6].

Decentralisation which started in 1999 saw responsibility for health education shift to the Ministry of Education, and led to a large scale expansion in midwifery training institutions. By 2012 there were over 200,000 midwives in Indonesia. Despite this, retention of midwives in rural areas continues to be a problem [2, 5, 7].

In 2001 the Ministry of Health adopted the World Health Organisation's Making Pregnancy Safer approach as a national strategy [8]. The new direction for Indonesia placed greater focus on complication prevention and birth preparedness [5]. There was also a gradual adoption of a set of universal minimum service standards for care, aimed at ensuring quality and accountability in primary health care service provision across the districts and municipalities [9]. This evolved into a Ministry of Health Decree (Decree 741/2008) that by 2015 the following goals would be met: 95% antenatal care coverage; 80% of obstetric complications attended; 90% of deliveries attended by skilled health providers with obstetric competencies; 90% post-partum visit coverage; and 100% coverage of basic health care and referral services for the poor.

A further Ministry of Health Decree (Decree 515/2011) launched the *Jampersal* maternity coverage scheme. The scheme was directed at the poorest 33% of the population, providing free basic maternal health care at local health centres (puskemas) and referral centres. This scheme was amalgamated into the National Health Insurance Scheme (*Jaminan Kesehatan Nasional*, JKN) in 2014 [10].

Despite the various iterations of national strategies, setting of targets, introduction of schemes, and large investments in expanded service provision, quality of and access to maternal health services remain at relatively low levels in Indonesia. The current reality for Indonesia is well short of the goal of universal access to health services that meet minimum standards. Reports by both the World Bank and the Indonesian National Academy of Sciences place a great deal of emphasis on the geographical inequities in access to health care services, including and especially higher level care and developing specific strategy for reaching the outer islands (non-Java) [2, 11].

The key points of concern are:

- deficiencies in coverage and large disparities in quality of services by region with rural areas being underserved [11];
- inadequate training of midwives [3];
- predominance of home-based care: the system of community-based midwifery provision promotes home-based care, which can be basic even with attendance by a SBA [7];
- Low coverage of emergency-level care. Indonesia has the lowest doctor-population ratio in South-East Asia [2]. The situation is even worse with specialists, with only 2,600 obstetricians nationally, compared to a goal of 35,000, with the majority of these working in Jakarta or other large urban centres in Java;
- Hierarchical referral system for maternal complications which creates potentially costly delays. The traditional birth attendant refers to the village midwife, who in turn refers to the Puskesmas, with referrals then going to the district hospital [5, 12, 13];
- Transport difficulties due to lack of ambulances and poor road infrastructure [11, 14].

S2 Supporting Information References.

1 Shiffman J. Generating Political Will for Safe Motherhood in Indonesia. *Social Science and Medicine* 2003, 56: 1197–207.
2 National Academy of Sciences. Reducing Maternal and Neonatal Mortality in Indonesia: Saving Lives, Saving the Future. Report of the Joint Committee on Reducing Maternal and Neonatal Mortality in Indonesia; Development, Security, and Cooperation; Policy and Global Affairs; National Research Council; Indonesian Academy of Sciences. Washington DC: National Acadamies Press, 2013.
3 Rokx C, Schieber G, Harimurti P, Tandon A, Somanathan A. Health Financing in Indonesia: A Reform Road Map. Directions in Development: Human Development. Washington DC: The World Bank, 2010.
4 Koblinsky M “Indonesia, 1990–1999” in: Koblinsky M. (ed.), *Reducing Maternal Mortality: Learning from Bolivia, China, Egypt, Honduras, Indonesia, Jamaica, and Zimbabwe*. Washington DC: The World Bank Human Development Network, 2003.
5 World Bank “…and then she died” Indonesia Maternal Health Assessment, World Bank Report 53327, Jakarta: World Bank, 2010.
6 Hill S, Goeman L, Sofiarini R, Djara M. `Desa SIAGA’, the ‘Alert Village’: the Evolution of an Iconic Brand in Indonesian Public Health Strategies. *Health Policy and Planning* 2013, 1-12.
7 Makowiecka K, Achadi E, Izati Y, Ronsmans C. Midwifery Provision in Two Districts in Indonesia: How Well are Rural Areas Served? *Health Policy and Planning* 2008, 23: 67–75.
8 Nayoan C. Childbirth and Maternal Health in Indonesia. *MKM* 2011, 6(1).
9 Hayes A, Harahap N. What is Ailing the Indonesian Health System? Governance, National Policy and the Poor. In Chris Manning & Sudarno Sumarto (eds.), Employment, Living Standards and Poverty in Contemporary Indonesia. Singapore: Institute of Southeast Asian Studies (ISEAS), 2011, pp. 206-220.
10 Achadi E, Achadi A, Pambudi E, Marzoeki P. A Study on the Implementation of Jampersal Policy in Indonesia, World Bank Health, Nutrition, and Population (HNP) Discussion Paper 91325, September 2014. https://openknowledge.worldbank.org/handle/10986/20740. (Accessed 27 April 2018.)
11 World Bank. Universal Maternal Health Coverage? Assessing the Readiness of Public Health Facilities to Provide Maternal Health Care in Indonesia. Jakarta: World Bank Report No. 90510-ID, 2014.
12 Nasir S, Ahmed R, Kurniasari M et al. Context Analysis: Close-To-Community Maternal Health Providers in South West Sumba and Cianjur, Indonesia. Jakarta: Reachout, 2014.
13 D'Ambruoso L, Byass P, Qomariyah S. Maybe it was Her Fate and Maybe She Ran Out of Blood: Final Caregivers' Perspectives on Access to Care in Obstetric Emergencies in Rural Indonesia. *Journal of Biosocial Science* 2010, 42: 213-241.
14 Kowalewski M, Jahn A. Health Professionals for Maternity Services: Experiences on Covering the Population with Quality Maternity Care. *Studies in Health Services Organisation and Policy* 2001, 17(2): 131-148.
