# Supplementary material for: Understanding the determinants of maternal mortality: An observational study using the Indonesian Population Census
Source: PLoS One. 2019 Jun 3;14(6):e0217386. doi: 10.1371/journal.pone.0217386 (PMC6546237; doi:10.1371/journal.pone.0217386)

S3 Appendix. Additional results

Table A reports maternal mortality ratios and related variables by province.

Table B reports unadjusted results from logistic regressions.

Table C reports results when we include region fixed effects (where regions are defined as Java and Bali; Sumatra, Kalimantan, Sulawesi, East and West Nusatenggara, Maluku and Papua). The results are largely unaffected.

Figs A and B are maps of Indonesia showing the geographic distribution of average distance to a hospital and the number of maternal deaths, respectively.

Table A: Maternal mortality by province

Province	*MMR (/100,000 live births)*	*Number of deaths*	*Average distance to a hospital*	*Number of midwives at village health post*	*Household head years of education*	*Population Density*	*Agriculture as main economic activity*
Aceh	121.60	161	17.36	0.19	8.92	78	0.80
North Sumatra	144.35	551	13.33	0.63	9.19	179	0.68
West Sumatra	181.61	66	2.90	0.47	10.12	116	0.40
Riau	115.21	196	18.39	0.57	8.99	64	0.66
Jambi	149.77	130	21.48	0.37	8.29	62	0.78
South Sumatra	93.72	191	21.48	0.88	8.19	82	0.71
Bengkulu	131.53	62	18.80	0.25	8.55	86	0.79
Lampung	116.04	237	17.32	0.72	8.21	220	0.83
Bangka-Belitung	172.64	57	20.39	0.65	7.87	75	0.54
Riau Island	84.10	45	7.71	0.28	10.08	206	0.20
Jakarta	78.43	179	0.51	0.00	10.69	14518	0.01
West Java	114.08	1208	9.21	0.25	8.70	1222	0.55
Central Java	123.99	928	7.59	0.75	7.72	989	0.76
Yogyakarta	109.18	77	3.77	0.47	9.67	1107	0.67
East Java	135.92	1043	10.02	0.43	7.95	786	0.73
Banten	96.97	271	8.58	0.16	8.66	1106	0.43
Bali	46.94	41	9.02	0.49	9.27	676	0.54
West Nusa Tenggara	127.52	173	18.91	0.48	7.58	243	0.85
East Nusa Tenggara	340.19	495	28.31	0.11	6.69	97	0.89
West Kalimantan	133.20	158	26.45	0.41	7.24	30	0.75
Central Kalimantan	243.05	147	26.43	0.25	8.45	14	0.71
South Kalimantan	164.19	162	15.06	0.63	8.23	94	0.62
East Kalimantan	101.90	100	13.84	0.05	9.30	17	0.43
North Sulawesi	235.77	119	13.70	0.17	8.99	164	0.71
Central Sulawesi	332.51	245	29.01	0.58	7.87	43	0.81
South Sulawesi	158.87	336	14.89	0.43	7.63	173	0.71
Southeast Sulawesi	206.48	150	27.36	0.18	7.96	59	0.75
Gorontalo	371.22	95	24.00	0.27	6.73	93	0.80
West Sulawesi	338.48	121	28.09	0.49	7.03	69	0.88
Maluku	171.46	69	22.89	0.40	8.67	33	0.74
North Maluku	176.90	54	22.68	0.13	8.31	33	0.80
West Papua	280.52	64	14.39	0.00	9.05	8	0.54
Papua	220.64	144	19.36	0.03	7.03	9	0.72

Note that there are no midwives at village health posts in Jakarta because there are no village health posts in the capital city. The population density figures are for 2010 from the Indonesian Statistical Agency. Available at: https://www.bps.go.id/dynamictable/2015/09/07/842/kepadatan-penduduk-menurut-provinsi-2000-2015.html Accessed on 14/06/2018.

Table B. Unadjusted Logistic Regression results. Dep. Variable – Maternal Death

VARIABLES	(1)<br>Odds ratio	(2)<br>95% CI		
Age	0.954	0.931	-	0.978
Age squared	1.002	1.001	-	1.002
**Household Head's characteristics:**				
Highest level of education attained:				
Primary school	0.620	0.583	-	0.660
Junior high school	0.397	0.366	-	0.430
Senior high school or above	0.326	0.304	-	0.350
Employed	0.610	0.566	-	0.657
**Household Characteristics**				
Urban	0.719	0.684	-	0.755
Poor quality floor	1.359	1.289	-	1.433
Doesn't have a toilet	1.423	1.345	-	1.504
**Village Characteristics**				
Most households have unimproved water source	1.064	1.031	-	1.098
Most households do not have a toilet	1.312	1.225	-	1.406
Main source of income - agriculture	1.448	1.371	-	1.529
Widest road surface is unpaved	1.337	1.190	-	1.501
Main road cannot be passed all year round	1.502	1.334	-	1.692
**Health Service Access**				
Distance to the nearest hospital (10 kms)	1.093	1.081	-	1.106
Distance to the nearest health centre (10 kms)	1.178	1.140	-	1.218
No. doctors working at health centre	0.911	0.893	-	0.930
No. midwives working at health centre	0.991	0.987	-	0.995
Health centre has inpatients	1.016	0.966	-	1.067
No. of doctors living in the village	0.971	0.964	-	0.978
No. of midwives working in the village health post	0.950	0.916	-	0.986
Village has a birthing station	1.043	0.989	-	1.099
Birthing station has an inpatients facility	0.934	0.843	-	1.035

Note: We present results of univariate logistic regressions. Exceptions are that age and age squared are included as explanatory variables in a single model. We also include all education indicators in a single model. For household head’s education, the base category is no education or incomplete primary school education. Standard errors are clustered at the village level.

Table C: Logistic Regressions including regional fixed effects.
Dependent variable – Maternal death (0/1).

	(1)	(2)
VARIABLES	odds ratio	95% CI
**Woman's characteristics**		
Age	1.025	1.000 - 1.052
Age squared	1.000	1.000 - 1.001
**Household Head's characteristics:**		
Highest level of education attained:		
Primary school	0.681	0.640 - 0.726
Junior high school	0.451	0.415 - 0.490
Senior high school or above	0.365	0.337 - 0.394
Employed	0.619	0.573 - 0.667
**Household Characteristics**		
Urban	1.041	0.969 - 1.119
Poor quality floor	0.968	0.912 - 1.028
Doesn't have a toilet	1.040	0.977 - 1.107
**Village Characteristics**		
Most households have unimproved water source	1.004	0.966 - 1.043
Most households do not have a toilet	0.985	0.913 - 1.063
Main source of income - agriculture	1.045	0.965 - 1.132
Widest road surface is unpaved	0.974	0.856 - 1.108
Main road cannot be passed all year round	1.049	0.916 - 1.201
**Health Service Access**		
Distance to the nearest hospital (10 kms)	1.024	1.008 - 1.040
Distance to the nearest health centre (10 kms)	0.992	0.945 - 1.041
No. doctors working at health centre	0.976	0.957 - 0.994
No. midwives working at health centre	1.000	0.997 - 1.004
Health centre has inpatients	0.963	0.914 - 1.015
No. of doctors living in the village	0.992	0.986 - 0.998
No. of midwives working in the village health post	0.959	0.920 - 0.999
Village has a birthing station	0.996	0.936 - 1.061
Birthing station has an inpatients facility	0.855	0.765 - 0.956
Region Effects		
Sumatra	1.153	1.079 - 1.231
Sulawesi	1.626	1.494 - 1.771
Kalimantan	1.250	1.121 - 1.393
East and West Nusa Tenggara/Maluku/Papua	1.582	1.439 - 1.738
Constant	0.00111	0.000735 - 0.00168
Observations	5,567,029	

Notes: Standard errors are clustered at the village level. For household head education, the base category is no education or incomplete primary school. For the regions, the base category is Java-Bali.

Figure A. Average distance to the hospital in kilometres by province

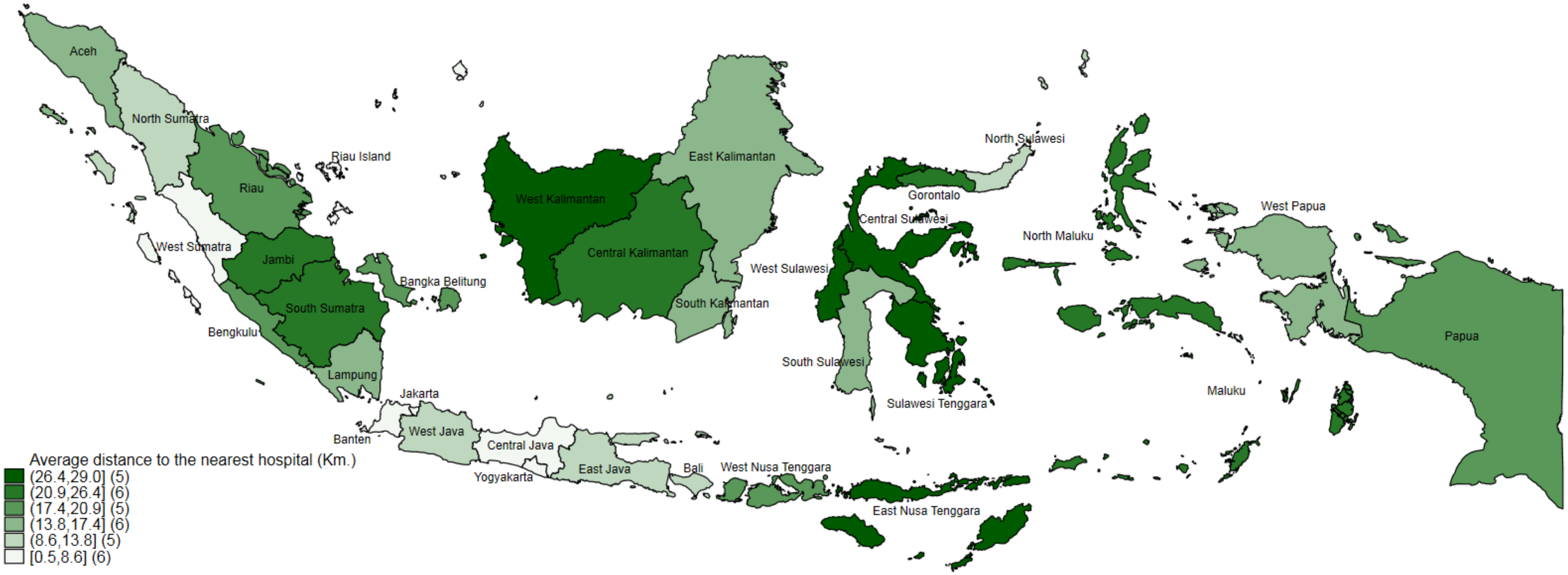

Figure B. Maternal death counts by province

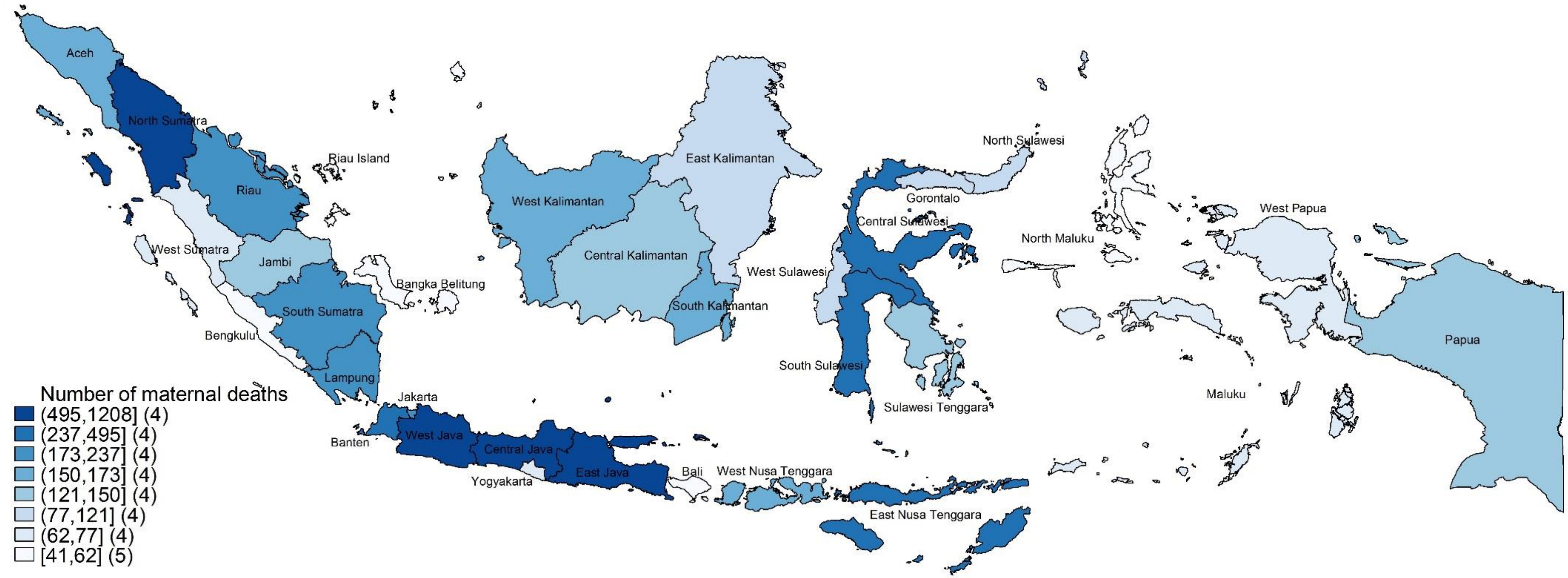

Supplement: S3 Appendix — (PDF) [file pone.0217386.s003.pdf]
