# Supplementary material for: Understanding the determinants of maternal mortality: An observational study using the Indonesian Population Census
Source: PLoS One. 2019 Jun 3;14(6):e0217386. doi: 10.1371/journal.pone.0217386 (PMC6546237; doi:10.1371/journal.pone.0217386)
Supplement: S4 Appendix — (PDF) [file pone.0217386.s004.pdf]

S4 Appendix. Indonesia's maternal mortality ratio

Calculating the Indonesian mortality ratio is complicated by the fact that there is not reliable civil registration and death reporting. The Indonesian government nominated the Indonesian Demographic and Health Survey as the data source from which to calculate the country's MMR for the Millennium Development Goals. Fig A plots point estimates of Indonesia's maternal mortality ratio between 1990 and 2012 from the official Demographic and Health Survey (DHS) reports. The Demographic and Health Survey allows for calculation of estimates over the five-year period prior to and including the survey year. Prior to 2012, these estimates indicate Indonesia was making moderate progress. The latest result though (2012), produced an alarming point estimate of 359 deaths per 100,000 live births, suggesting very little overall progress since 1990, and shifting Indonesia back into the "high" maternal mortality category.

There are issues with measurement of MMRs using the DHS data sets that cause us to qualify the message apparent in Fig A. The most crucial step in estimating the MMR is identifying maternal deaths. The DHS relies on the 'sisterhood method'. The DHS women's questionnaire asks each woman in the household about the survivorship of all the live-born children of her mother, that is, her maternal siblings. For any female siblings who died at age 10 or older, further questions determine whether the death was pregnancy-related (pregnant when died, died during childbirth or died within 2 months after the end of a pregnancy). The sisterhood method allows data to be collected on pregnancy-related deaths for a larger sample of women – not just those in the immediate household being surveyed, but also the sisters of those women in the surveyed household - however, even then the number of deaths captures is small. The 2012 DHS figure for Indonesia was based on only 92 reported maternal deaths [1]. As a result, these point estimates of the MMR carry such large sampling errors that it is not possible to determine whether Indonesia's maternal mortality rate has indeed increased. Even the improvement of the MMR from 390 in 1990-94 to 228 in 2003-07 is not a statistically significant decline [2].

An alternative to the DHS is to use a model-based approach which models the MMR drawing on a variety of data sources and combines these in a coherent way to provide estimates of MMRs. This is the approach used by the Maternal Mortality Estimation Inter-Agency Group (MMEIG), a group comprised of representatives from the WHO, UNICEF, UNFPA, UNPD, World Bank, National University of Singapore and the University of California at Berkeley [3]. On this basis Indonesia has been making progress in reducing its MMR from 446 in 1990 to 165 in 2010 to 126 in 2015 (a decrease of 72% since 1990).

Fig B plots Indonesia's progress using this method relative to other countries in the region. It is important to understand that the MMEIG estimates for Indonesia are at least in part constructed from a multi-level regression model which uses variables such as country GDP, general fertility rates and rates of skilled attendants at birth to predict the proportion of deaths among women of reproductive age that are due to maternal causes. This figure is then converted to a MMR by incorporating data on population estimates and live births so the estimate for 2010 of 165 is best thought of as a model projection of what ought to have occurred, given the values of these inputs. It takes no account of the myriad other factors that would cause variation from the model. For example, with Indonesia's very large and geographically spread population, predicting an improvement in the MMR based on increased numbers of birth attendants will fail to capture the potential variation in spatial distribution of these attendants, or the variable quality of their training, or their access to the required equipment or medical supplies in the health centres where they work. Being a model based on slowly evolving input variables, the MMEIG model is also likely to predict a continuation of the trend evident in past data.

Finally, the 2010 Population Census data can be used to construct an estimate of Indonesia's MMR. There are a number of challenges associated with estimating MMRs from census data [4,5]. As discussed in the main text, the MMR implied from the raw census data of 8075 maternal deaths and 5,866,791 live births, is 137 deaths per 100,000 live births. Adjusting the raw estimate to correct for potential underreporting of pregnancy-related deaths produces an MMR of 263 [6]. Because the critical questions are not used in previous censuses, there is no capacity to compare the same measure across time.

So for 2010 we have estimates of the MMR ranging from 137 using the raw Population Census data, to 165 using the model-based MMEIG method, to 263 using the census data and correcting for potential under-reporting of deaths, to 359 using the DHS sisterhood method. The two series of estimates that allow a comparison across time show very different changes between 1990 and 2010. The DHS suggests a decline of only 8%, whereas the MMEIG figures suggest a decrease of 52% over the same period.

Figure A. Maternal Mortality Ratio Estimates for Indonesia

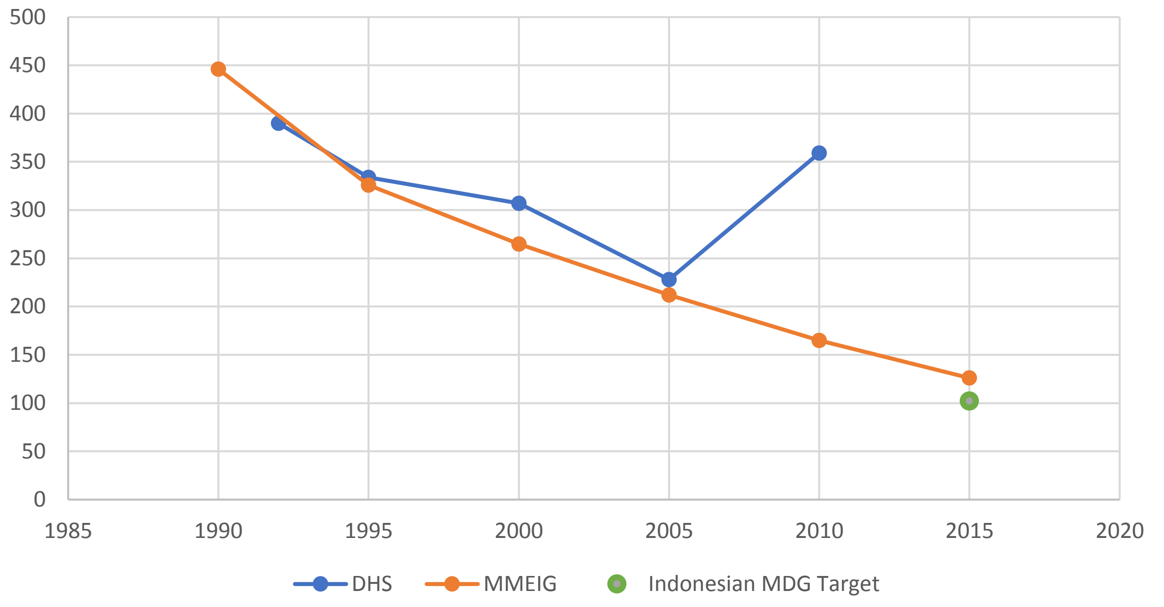

Figure B. Trends in Maternal Mortality Ratios. South East Asian Countries, 1990-2015

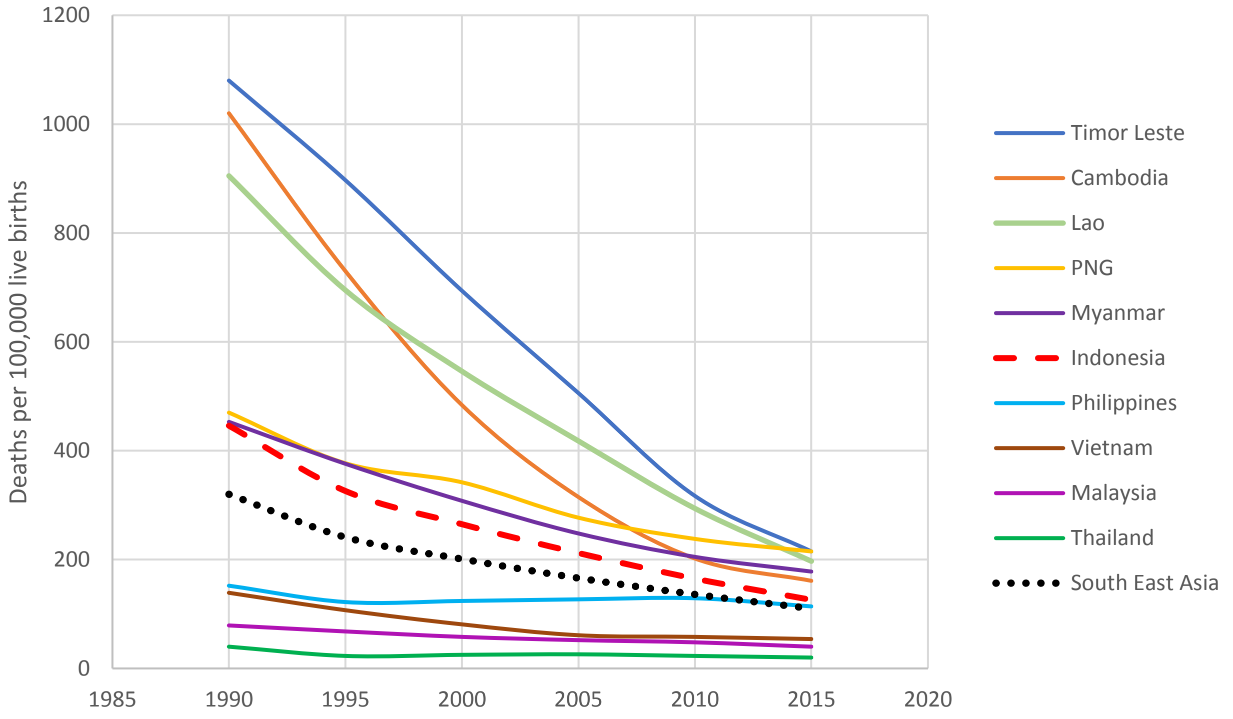

Previous Literature on Determinants of Maternal Mortality in Indonesia

We searched PubMed for studies with the search string "("maternal mortality" OR "maternal death") in the Title AND ("Indonesia") in the Title/Abstract" which were published between 2000 and February 2018. Of the 18 studies that met these criteria, nine examined the determinants of maternal mortality (the others being commentary pieces, focused on measuring maternal mortality rates or evaluations of a specific program's impact on

determinants of maternal health). Two studies used verbal autopsies to investigate causes of maternal death. One investigated causes of maternal deaths in two rural districts in Java and found that delays in seeking and receiving care were often reported by family members to have occurred [7]. The other examined 130 maternal deaths in Kalimantan and identified delays in decision-making and poor quality of care as contributing factors [8]. Of the seven quantitative studies of causes of maternal death, four studies were retrospective case-control studies based on relatively small samples from hospitals in cities in Java [9 – 12]. Only one of these studies examined determinants of maternal death beyond direct medical causes, finding that living outside the city and incomplete attendance at ante-natal visits were significant determinants of maternal mortality [9]. Two studies used data from a population-based case-control study in two districts on Java. They found that the odds of dying increased with distance to a health centre and among women who were assisted by a health professional [13], mortality rates were very high among poor women [14]. One study used data from the Demographic and Health Surveys and found a very strong positive association between poverty and maternal death in Indonesia [15].

S4 Appendix References.

1 Statistics Indonesia. *Indonesia Demographic and Health Survey 2012 Final Report*, FR275. Jakarta: Statistics Indonesia, 2013.

2 Statistics Indonesia. *Indonesia Demographic and Health Survey 2007 Final Report*, FR218. Jakarta: Statistics Indonesia, 2008.

3 WHO. Trends in Maternal Mortality: 1990 to 2013 Estimates by WHO, UNICEF, UNFPA, The World Bank and the United Nations Population Division. Geneva: World Health Organization, 2013.

4 Hill K, Stanton C. Measuring maternal mortality through the census: rapier or bludgeon? *Journal of Population Research* 2011 28(1):31–47.

5 Leone T. Measuring Differential Maternal Mortality Using Census Data in Developing Countries. *Population, Space and Place* 2014, 20:581-591.

6 Soemantri S. Mortality Data Quality. Paper presented at Workshop and Training on SP 2010: Estimation of MMR, Aston Hotel. Bogor: November 12-14, 2012. As cited in National Research Council. Reducing Maternal and Neonatal Mortality in Indonesia: Saving Lives, Saving the Future. National Acadamies Press, Washington DC, 2013. doi:0.17226/18437.

7 D'Ambruso L, Byass P, Qomariyah SN, Ouedraogo, M. A lost cause? Extending verbal autopsy to investigate biomedical and socio-cultural causes of maternal death in Burkina Faso and Indonesia. Soc Sci Med. 2010 Nov;71(10):1728-38. doi: 10.1016/j.socscimed.2010.05.023. Epub 2010 Jun 4.

8 Supratikto G, Wirth ME, Achadi E, Cohen S, Ronsmans C. A district-based audit of the causes and circumstance of maternal deaths in South Kalimantan, Indonesia. Bull World Health Organ. 2002;80(3):228-34.

9 Taguchi N, Kawabata M, Maekawa M, Maruo T, Aditiawarman, Dewata L Influence of socio-economic background and ante-natal care programmes on maternal mortality in Surabaya, Indonesia. Trop Med Int Health. 2003 Sep;8(9):847-52.

10 Mawarti Y, Utarini A, Hakimi, M. Maternal care quality in near miss and maternal mortality in an academic public tertiary hospital in Yogyakarta, Indonesia: a retrospective cohort study. BMC Pregnancy Childbirth. 2017 May 22;17(1):149. doi: 10.1186/s12884-017-1326-4.

11 Akbar A, Wicaksono B, Dachlan EG. Maternal mortality and its mainly possible cause pre-eclampsia/eclampsia in developing country (Surabaya - Indonesia as the model). Pregnancy Hypertens. 2012 Jul;2(3):184. doi: 10.1016/j.preghy.2012.04.019. Epub 2012 Jun 13.

12 Akbar A, Laksana MA, Dachlan EG, Prasetyo B. Maternal death risk factor score based on hospital reference pattern and maternal condition of eclamptic woman in Soetomo Hospital, Surabaya, Indonesia. Pregnancy Hypertens. 2012 Jul;2(3):183. doi: 10.1016/j.preghy.2012.04.018. Epub 2012 Jun 13.

13 Scott S, Chowdhury ME, Pambudi, ES, Qomariyah SN, Ronsmans C. Maternal mortality, birth with a health professional and distance to obstetric care in Indonesia and Bangladesh.Trop Med Int Health. 2013 Oct;18(10):1193-201. doi: 10.1111/tmi.12175.

14 Ronsmans C, Scott S, Qomariyah SN, Achadi E, Braunholtz D, Marshall T, Pambudi E, Witten KH, Graham WJ Professional assistance during birth and maternal mortality in two Indonesian districts. Bull World Health Organ. 2009 Jun;87(6):416-23.

15 Graham WJ, Fitzmaurice AE, Bell JS, Cairns JA. The familial technique for linking maternal death with poverty. Lancet. 2004 Jan 3;363(9402):23-7.
