# Supplementary material for: Understanding the determinants of maternal mortality: An observational study using the Indonesian Population Census
Source: PLoS One. 2019 Jun 3;14(6):e0217386. doi: 10.1371/journal.pone.0217386 (PMC6546237; doi:10.1371/journal.pone.0217386)
Supplement: S1 Checklist — (PDF) [file pone.0217386.s005.pdf]

S1 Checklist. STROBE Statement

—Checklist of items that should be included in reports of *cross-sectional studies*

	Item No	Recommendation	Location
**Title and abstract**	1	(*a*) Indicate the study's design with a commonly used term in the title or the abstract	Title
		(*b*) Provide in the abstract an informative and balanced summary of what was done and what was found	Abstract – paragraphs 2-3.
**Introduction**			
Background/rationale	2	Explain the scientific background and rationale for the investigation being reported	Introduction paragraphs 1-2
Objectives	3	State specific objectives, including any prespecified hypotheses	Introduction paragraph 3
**Methods**			
Study design	4	Present key elements of study design early in the paper	Introduction paragraph 3, Methods - Data Sources; Statistical Analysis; Covariates subsections.
Setting	5	Describe the setting, locations, and relevant dates, including periods of recruitment, exposure, follow-up, and data collection	Methods – Data Sources; Structure of the Indonesian Health Sector sub-sections.
Participants	6	(*a*) Give the eligibility criteria, and the sources and methods of selection of participants	Methods paragraph 2.
Variables	7	Clearly define all outcomes, exposures, predictors, potential confounders, and effect modifiers. Give diagnostic criteria, if applicable	Methods paragraph 2 & Covariates sub-section; S1 Appendix.
Data sources/ measurement	8*	For each variable of interest, give sources of data and details of methods of assessment (measurement). Describe comparability of assessment methods if there is more than one group	S1 Appendix, as referenced in Methods paragraph 1.
Bias	9	Describe any efforts to address potential sources of bias	Methods - Statistical Analysis sub-section.
Study size	10	Explain how the study size was arrived at	Methods - Covariates subsection; S1 Appendix, paragraphs 4 and 5.
Quantitative variables	11	Explain how quantitative variables were handled in the analyses. If applicable, describe which groupings were chosen and why	Methods - Covariates sub-section.

Statistical methods	12	(*a*) Describe all statistical methods, including those used to control for confounding	Methods - Statistical Analysis sub-section.
		(*b*) Describe any methods used to examine subgroups and interactions	Methods - Statistical Analysis sub-section.
		(*c*) Explain how missing data were addressed	Methods – Covariates subsection.
		(*d*) If applicable, describe analytical methods taking account of sampling strategy	NA
		(*e*) Describe any sensitivity analyses	Methods - Statistical Analysis sub-section.
**Results**			
Participants	13*	(a) Report numbers of individuals at each stage of study—eg numbers potentially eligible, examined for eligibility, confirmed eligible, included in the study, completing follow-up, and analysed	Methods – Covariates subsection.
		(b) Give reasons for non-participation at each stage	NA
		(c) Consider use of a flow diagram	NA
Descriptive data	14*	(a) Give characteristics of study participants (eg demographic, clinical, social) and information on exposures and potential confounders	Table 1
		(b) Indicate number of participants with missing data for each variable of interest	S1 Appendix, paragraph 5.
Outcome data	15*	Report numbers of outcome events or summary measures	Table 1
Main results	16	(*a*) Give unadjusted estimates and, if applicable, confounder-adjusted estimates and their precision (eg, 95% confidence interval). Make clear which confounders were adjusted for and why they were included	Table 2. S3 Appendix, Table S3.2.
		(*b*) Report category boundaries when continuous variables were categorized	NA
		(*c*) If relevant, consider translating estimates of relative risk into absolute risk for a meaningful time period	Results, Determinants of maternal mortality, paragraphs 3 & 4.
Other analyses	17	Report other analyses done—eg analyses of subgroups and interactions, and sensitivity analyses	Results, Determinants of maternal mortality, paragraph 5. Table S3.3.
**Discussion**			
Key results	18	Summarise key results with reference to study objectives	Discussion paragraph 1
Limitations	19	Discuss limitations of the study, taking into account sources of potential bias or imprecision. Discuss both	Discussion paragraph 3

		direction and magnitude of any potential bias	
Interpretation	20	Give a cautious overall interpretation of results considering objectives, limitations, multiplicity of analyses, results from similar studies, and other relevant evidence	Discussion paragraph 2.
Generalisability	21	Discuss the generalisability (external validity) of the study results	Discussion paragraph 4.
**Other information**			
Funding	22	Give the source of funding and the role of the funders for the present study and, if applicable, for the original study on which the present article is based	Detailed in the additional submission required information.

*Give information separately for exposed and unexposed groups.
